# Whey Protein Hydrolysate Renovates Age-Related and Scopolamine-Induced Cognitive Impairment

**DOI:** 10.3390/nu15051228

**Published:** 2023-02-28

**Authors:** Ning Ding, Hanxiu Meng, Chao Wu, Wallace Yokoyama, Hui Hong, Yongkang Luo, Yuqing Tan

**Affiliations:** 1 Beijing Laboratory for Food Quality and Safety, College of Food Science and Nutritional Engineering, China Agricultural University, Beijing 100083, China; 2 Research and Development, Hilmar Cheese Company, Hilmar, CA 95324, USA; 3 Healthy Processed Foods Research Unit, Agricultural Research Service, United States Department of Agriculture, Albany, CA 94710, USA

**Keywords:** whey protein hydrolysate, cognitive impairment, hippocampus histopathology

## Abstract

Whey protein and its hydrolysates are ubiquitously applied in the food system. However, their effect on cognitive impairment remains unclear. This study aimed to investigate the potential ability of whey protein hydrolysate (WPH) to ameliorate cognitive degeneration. WPH intervention in Crl:CD1 (ICR, Institute for cancer research) mice and aged C57BL/6J mice in a scopolamine-induced cognitive impairment model for 10 days were evaluated. Behavioral tests indicated that WPH intervention improved the cognitive abilities in ICR and aged C57BL/6J mice (*p* < 0.05). Scopolamine enhanced the A*β*_1-42_ level in the brain tissue, and the WPH intervention exhibited a similar therapeutic effect to donepezil in ICR mice. A noticeable reduction occurred in serum A*β*_1-42_ level of aged mice treated with WPH. The histopathological study of the hippocampus showed that WPH intervention alleviates neuronal damage. Hippocampus proteomic analysis suggested possible mechanisms of WPH action. The relative abundance of *Christensenellaceae,* a gut microbe related to Alzheimer’s disease, was altered by WPH intervention. This study demonstrated that short-term WPH intake protected against memory impairment induced by scopolamine and aging.

## 1. Introduction

As the global population ages, dementia associated with aging is increasing. Alterations in the brain and the ultimate loss of neurons, especially in the frontal cortex and hippocampus, occur with aging [1,2]. Alzheimer’s disease (AD) is widely recognized to be an age-related neurodegenerative disease. Recent data from the 2021 Alzheimer’s World Report indicated that more than 55 million people worldwide have dementia [3]. About 60% of those dementia patients lived in low- and middle-income countries. Meanwhile, China, India, and their South Asian and Western Pacific neighbors have the fastest growing rate of elderly populations. With the aging demographic, increasing neurological disorders are more distressing and are placing families and society under tremendous economic and psychological pressures. Treatments for AD and other dementias should be a global health priority [4,5].

Effective pharmacological therapy for cognitive decline has remained elusive since 2003, especially after the recent controversy over the efficacy, side effects, and price of the first novel medicine approved by the FDA (Food and Drug Administration) in 2021 [6]. Therefore, the focus of clinical care has shifted from medicinal development to disease intervention. Emerging evidence suggests that lifestyle-related factors such as dietary habits and physical and mental activities are associated with cognitive performance [7,8]. It is plausible that dietary supplements have an interventional effect on dementia. Consequently, there have been over 1000 research publications on nutritional supplements with neuroprotective and cognitive-enhancing effects since 2000. Various dietary supplements and food items (omega-3, polyunsaturated fatty acid, vitamin D, flavonoids, etc.) have been investigated for their protective effect against AD. Among these substances used for nutritional supplementation, bioactive peptides, such as peptides from dairy products and fish protein hydrolysates, have gained significant interest as possible modified protective factors to alleviate memory problems. Protein hydrolysates of Tilapia heads have been proven to have neuroprotective effects, which could normalize the cholinergic and oxidative stress system in BALB/c mice via the microbiota–gut–brain axis [9]. In particular, it is reported that dairy product consumption is highly associated with better cognitive performance [10], which suggests that specific components such as bioactive peptides may be beneficial for brain function. Increasing evidence suggested that plant-derived peptides promote brain health and may reduce the risk of cognitive impairment, by alleviation of oxidative damage, anti-inflammatory action, and beneficial gut microbiome alteration [11,12].

Whey protein (WP) is a byproduct generated in the process of making cheese, casein, and other dairy products. WP contains β-lactoglobulin, α-lactalbumin, immunoglobulins, and essential amino acids [13]. WP is ubiquitously applied in food industries. It is readily available and economical. Whey protein isolates and hydrolysates have been well known for their positive effects on muscle anabolism, metabolic equilibrium, intestinal health, blood pressure, blood glucose, and lipid levels [14,15]. Studies have shown peptides obtained from whey protein by specific enzymatic hydrolysis, such as tryptophan–tyrosine (WY)-related peptides, especially the glycine–threonine–tryptophan–tyrosine (GTWY) peptide, have some features in brain function improvement [16]. However, the antioxidant and neuroprotective properties of whey protein hydrolysates (WPH) in aging individuals have not been widely elucidated. Therefore, we performed a short-term dietary intervention of WPH rich in GTWY peptide in two mice models to explore its capacity to alleviate age-related cognitive decline and its possible mechanism.

## 2. Materials and Methods

### 2.1. Chemicals

WPH (Hilmar PROtelyze™ Bio-Brain) was kindly donated by Hilmar Cheese Co., Ltd. (Hilmar, CA, USA). Donepezil and scopolamine were obtained from Sigma-Aldrich (Beijing, China). The acetylcholinesterase (AchE) assay kit was purchased from Solarbio Science & Technology Co., Ltd. (Beijing, China). A*β*_1-42_ (Amyloid Beta 1-42) Elisa kit, superoxide dismutase (SOD) assay kit, and malondialdehyde (MDA) assay kit were obtained from Nanjing Jiancheng Bioengineering Institute (Nanjing, China). All other chemical reagents were of analytical grade.

### 2.2. Determination of Molecular Weight (MW) Distribution and Peptide Sequences in WPH

The MW distribution was determined according to Wang et al. [17] using size exclusion (SE)-HPLC with a TSK gel G2000 SWXL column (7.8 × 300 mm, TOSOH, Tokyo, Japan) for analysis. Peptide sequences of WPH were identified using liquid chromatography–tandem mass spectrometry (LC-MS/MS) and searched in the MASCOT 2.4 search engine (Matrix Science, Boston, MA, USA) based on UniProt Knowledgebase (UniProtKB) database. The result was quantified by calibration curve using AQUA peptide (Sigma Aldrich, Shanghai, China) as a standard.

### 2.3. Quantification of Peptide GTWY

The WPH powder was dissolved at 10 mg/mL. Labeled G(13C2,15N)TWY was mixed in citrate buffer to prepare a 200 µg/mL internal standard working solution. The standard working solution is 500 µg/mL GTWY in 10% citrate buffer. The sample was analyzed with Waters nano Acquity HPLC (Milford, MA, USA). The mobile phase constituents are shown in Supplementary Table S1.

### 2.4. Animals and Treatment

The experiment scheme is shown in Figure S1. All animal experiments were performed in accordance with the guidelines of the Animal Ethical Committee of China Agricultural University (Aw82202202-4-1). Male C57BL/6J mice aged 7 and 20 months and Crl: CD1 (ICR) mice aged 8 months were purchased from Wukong Biotechnology Co., LTD (Jiangsu, China). During the whole experiment, the mice were settled under standard conditions, with a 12 h light/dark cycle at 24 ± 1 °C and were freely allowed to maintenance feed (40.69% kcal protein, 20.34% kcal carbohydrate, 18.31% kcal fat, energy density 3.21 kcal/g, SPF Biotechnology Co., Ltd., Beijing, China, Permit number: SCXK (Jing) 2019-0010) and water. After one week of habituation, C57BL/6J mice (20-month-old) were randomly divided into different experimental groups (eight mice/group): the model group was given drinking water; the low- and high-dose WPH groups were given 10 and 100 mg/kg per day respectively; the WP group was treated with 100 mg/kg whey protein per day, and 7-month-old C57BL/6J mice given drinking water as the negative control group. The above treatment was carried out by gavage. ICR mice (eight mice/group) were randomly divided into the control, positive control, and WPH treatment groups by gavage of distilled water, donepezil hydrochloride, and WPH (100 mg/kg per day), respectively. Apart from the control group, memory impairment was induced by injection of scopolamine (0.85 mg/kg body weight per day) 40 min after gavage. The body weight and mean dietary intake were recorded every three days. The intragastric administration procedures lasted 10 days, followed by a one-week behavioral test. The mice were then euthanized, and serum and feces were collected. The visceral tissue was collected and immediately frozen with liquid nitrogen, and the hippocampus was fixed with a 4% (*v*/*v*) paraformaldehyde solution.

### 2.5. Behavioral Tests

During the behavioral test, mice in each group were given WPH or water by gavage 40 min before the testing and training. To avoid communication and interference between tested and untested mice, temporary segregation was necessary. At the end of the test for each mouse, it was transferred to a holding cage. Until all other mice in the same cage had been tested, they were put back into the home cage. All mice were subjected to the following three behavioral tests.

#### 2.5.1. Novel Object Recognition (NOR) Test 

This experiment was a relatively simple and efficient behavioral assay for evaluating mice’s exploration capabilities, recognition, and memory. The NOR test was performed in an empty open arena (40 × 25 × 20 cm), and the mice were placed in the center to acclimatization in advance. In the training stage, two identical objects (white squares with 4.5 cm sides) were stuck in the center of the arena, and the mouse was allowed to explore freely for 5 min. After 1 and 24 h, one of the identical objects was replaced with a new one (triangular pyramid with a height of 4.5 cm), and the mouse was allowed to explore in the same condition freely. The time spent exploring the old and new objects was recorded. The recognition index is calculated by dividing the time spent exploring new objects by the total exploration time.

#### 2.5.2. Shuttle Box Test

A shuttle box test was performed following the method of Tanichi et al. [18] with some modifications. Each mouse was put in the box on the training day to explore freely for 10 s. It was placed on the light side with the gate closed and exposed to electric shock (0.2 mA) of 10 s duration. One day after the training session, the avoidance task was performed. The number of times the mouse received an electric shock was recorded during the trial.

#### 2.5.3. Step-Down Avoidance Test

The memory and learning ability of mice were also investigated using step-down avoidance tasks according to the method of Rodrigues et al. [19]. During the training stage, the mice were lightly positioned in the rubber platform with the grid floor energized, and an electric foot shock (0.1 mA) was delivered when it stepped down, which was maintained for 8 min. After a day, the mice were again placed in the same place under energized conditions for 5 min, and the number of step-down times was recorded.

### 2.6. Determination of Biochemical Parameters

To separate serum, blood samples were centrifuged at 3000× *g* for 15 min at 4 °C. The activity of SOD and the content of MDA were determined following the corresponding reagent kits. The brain tissue was homogenized with 9 times the volume of PBS (phosphate buffered saline) and centrifuged at 12,000× *g* for 10 min to obtain supernatant. The levels of AchE and A*β*_1-42_ were determined using ELISA kits.

### 2.7. 16S rRNA Microbiome Sequencing

The microbial community genomic DNA of fecal samples was extracted individually via E.Z.N.A.^®^ soil DNA kit (Omega Bio-Tek, Norcross, GA, USA). NanoDrop 2000 (Thermo Fisher Scientific, Inc., Waltham, MA, USA) and 1% agarose gel electrophoresis were applied to assess the concentration and purity of the DNA extract. PCR amplification of the V3-V4 region in 16S rRNA was performed using primer pairs 338F (5′-ACTCCTACGGGAGGCAGCAG-3′) and 806R (5′-GGACTACHVGGGTWTCTAAT-3′). AxyPrep DNA Gel Extraction Kit (Axygen Bioscience, Union City, CA, USA) and QuantusTM Fluorometer (Promega, USA) were used to purify and quantified the PCR product. Purified amplicons were paired-end sequenced on an Illumina MiSeq PE300 platform (Illumina, San Diego, CA, USA) according to the manufacturer’s protocols by Majorbio Bio-Pharm Technology Co. Ltd. (Shanghai, China). Operational taxonomic units (OTUs) were clustered at a similarity level of 97% using UPARSE version 7.1. Based on the 16S rRNA database (e.g., Silva v138), each OTU representative sequence was analyzed and classified using RDP Classifier version 2.2. The raw data were deposited into the NCBI Sequence Read Archive (SRA) database with the accession number PRJNA903671.

### 2.8. Hematoxylin-Eosin (HE) Staining

The histopathological examination of the hippocampus was carried out with HE staining to assess morphological changes in the mouse hippocampus. The collected tissues were dehydrated with ethanol and fixed with paraffin. After being cut into slices, the samples were stained with hematoxylin and eosin. The microscopic images were digitized using a biological microscope (Nikon E400, Tokyo, Japan).

### 2.9. Proteomics Analysis

#### 2.9.1. Total Protein Extraction

The collected hippocampus tissue was mixed with lysis buffer (8 M urea and 1.0% SDS, containing protease inhibitor) and placed on ice for 30 min. After centrifugation, the supernatant was collected.

#### 2.9.2. Protein Digestion and Tandem Mass Tags (TMT) Labeling

TEAB (triethylammonium bicarbonate buffer) and TCEP (tris (2-carboxyethyl) phosphine) were mixed with samples and incubated for 60 min at 37 °C. Subsequently, IAM (Iodoacetamide) at a final concentration of 40 mM was added, and the mixture stood for 40 min at a temperature in dark conditions. After centrifugation for 20 min at 10,000× *g*, the precipitate was collected and dissolved by TEAB. Finally, the mixture was hydrolyzed overnight with trypsin at 37 °C.

Peptides were labeled with TMT reagent (A44522, Thermo Fisher Scientific, MA, USA). The samples were labeled, pooled, desalted, and vacuum-dried. The pooled samples were fractionated by ACQUITY UPLC BEH C18 Column (1.7 µm, 2.1 × 150 mm, Waters, Milford, MA, USA) on Vanquish Flex UPLC (Thermo Fisher Scientific, Waltham, MA, USA).

#### 2.9.3. LC-MS/MS Analysis

Evosep One system (Evosep, Denmark) combined with Obitrap Exploris 480 mass spectrometer (Thermo Fisher Scientific, Tewksbury, MA, USA) was used for LC-MS/MS analysis. The peptides were resolved and then separated on a C18 column (150 μm × 15 cm, Evosep, Denmark). In solvent A (0.1% formic acid in H_2_O), the linear elution gradient ranged from 5 to 90% for solvent B (0.1% formic acid in 80% acetonitrile) at 300 nL/min. The raw files were analyzed using Proteome Discovery v2.4 (Thermo Scientific, Tewksbury, MA, USA). 

#### 2.9.4. Bioinformatics Analysis

GO (Gene Ontology) was selected to perform GO annotation analysis, and KEGG (Kyoto encyclopedia of genes and genomes) enrichment analysis was used for DEPs characterization on the Majorbio Cloud Platform (www.majorbio.com, accessed on 18 November 2022).

### 2.10. Statistical Analysis

Quantitative data were represented as mean ± SD (standard deviation). The data were statistically processed with IBM SPSS 26.0 using Student’s *t* test to assess statistical significance. 

## 3. Results and Discussion

### 3.1. Composition and MW Distribution of WPH

**Figure 1:**
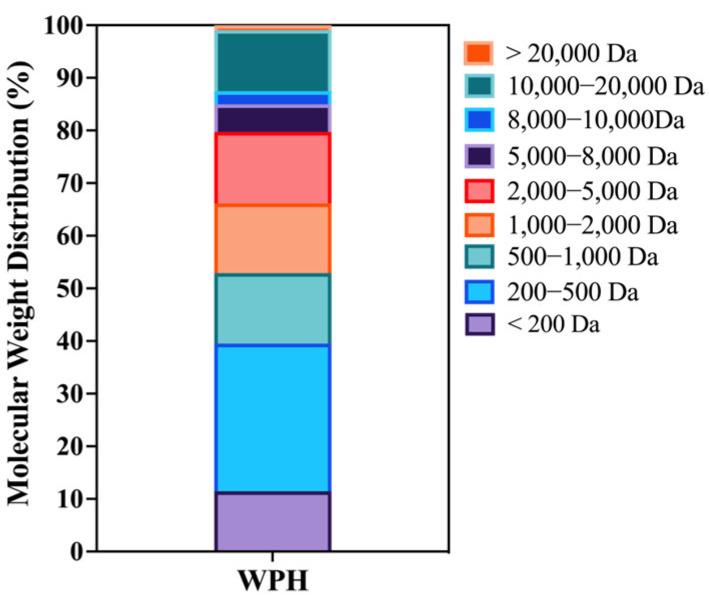
The molecular weight distribution of WPH.

As shown in Figure 1, the proportion of MW < 1 kDa fraction in WPH is 52.90%, while the content of the fraction with MW less than 5 kDa accounted for nearly 80%. As shown in the LC-MS/MS results in Figure S2, peptides derived from β-lactoglobulin make up the most significant proportion of WPH (54.75%). Moreover, KPTPEGDLEIL (sequence residues NO.47-57) is the most abundant peptide. The functional peptide GTWY is present in WPH at least 0.6%. It is generally accepted that peptide composition and molecular weight have a significant role in the functionality of protein hydrolysates [20]. In this study, the high content of peptides with low molecular weight probably contributes to its higher absorption and bioactivity.

### 3.2. Animal Metrics

**Figure 2:**
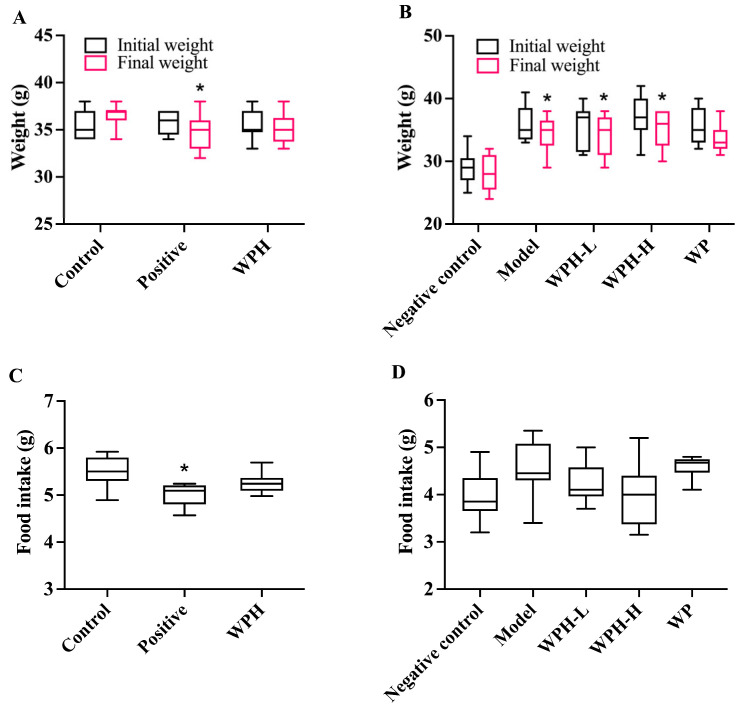
Effect of WPH on body weight of scopolamine-induced ICR mice (**A**) and aged C57BL/6J mice (**B**). Effect of WPH and WP on food intake in scopolamine-induced ICR mice (**C**) and aged C57BL/6J mice (**D**). * means *p* < 0.05 for the positive group vs. the control group. Control, positive, and WPH represent ICR mice treated with distilled water, donepezil hydrochloride, and whey protein hydrolysate (100 mg/kg), respectively. Model, WPH-L (low dose of whey protein hydrolysate), WPH-H (high dose of whey protein hydrolysate), and WP (whey protein) represent 20-month-old C57BL/6J mice treated with drinking water, 10 mg/kg whey protein hydrolysate, 100 mg/kg whey protein hydrolysate, and 100 mg/kg whey protein, respectively, and 7-month-old C57BL/6J mice were given drinking water as the negative control group.

The changes in mice body weights and food intake are shown in Figure 2. The considerable decrease in body weight and food intake of ICR mice in the positive control group was probably due to the donepezil injection (Figure 2A,C), which was similar to that of Yamamoto and coworkers [21]. In the absence of significant differences in food intake, the weight loss in 20-month-old mice may be due to a metabolic slowdown (Figure 2B,D). As illustrated in Table 1, the brain coefficients of C57BL/6J mice in the negative control group and model group show discernible differences due to age (*p* < 0.05), and significant variations were not observed between groups in terms of other organic coefficients. 

### 3.3. WPH Promoted the Spatial Memory and Cognitive Ability of Mice

**Figure 3:**
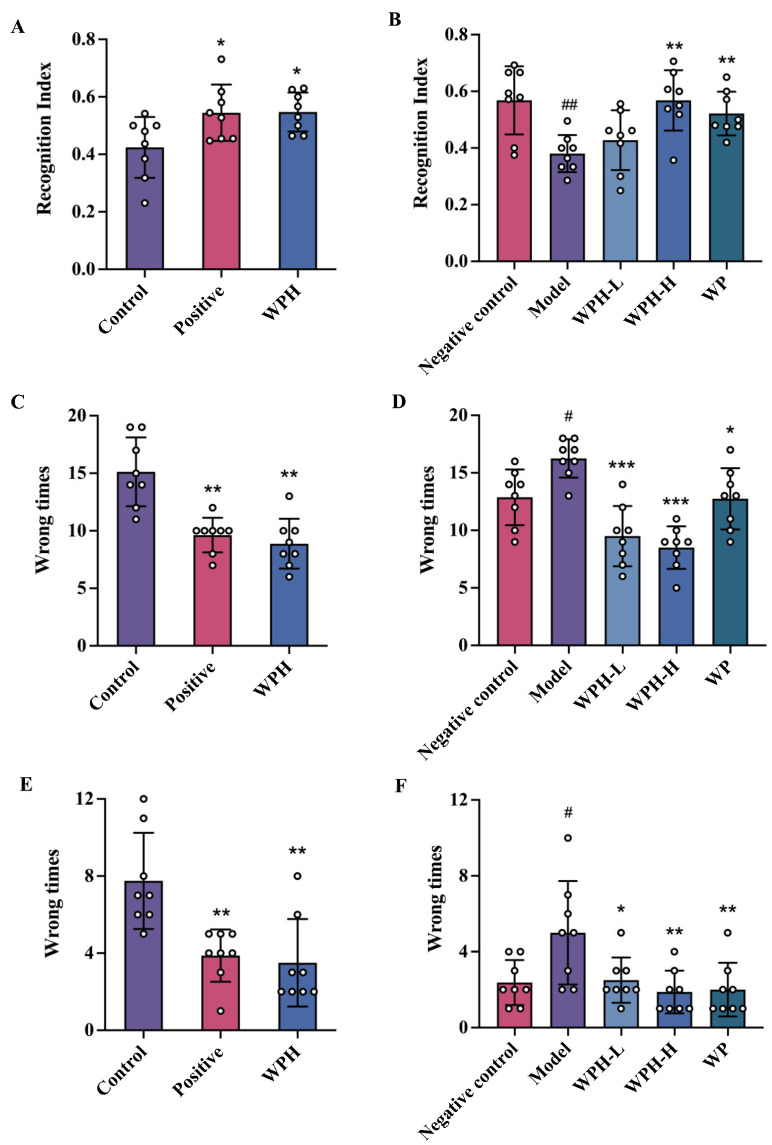
Effect of WPH on recognition index of NOR test of scopolamine-induced ICR mice (**A**) and old-aged C57BL/6J mice (**B**); shuttle box test of scopolamine-induced ICR mice (**C**) and old-aged C57BL/6J mice (**D**); step-down test of scopolamine-induced ICR mice (**E**) and old-aged C57BL/6J mice (**F**). ^#^ means *p* < 0.05, and ^##^ means *p* < 0.01 for model group vs. negative control group; *, ** and *** mean *p* < 0.05, <0.01 and <0.001 for other groups compared to the model group in C57BL/6J mice and the control group in ICR mice respectively. Control, positive, and WPH represent ICR mice treated with distilled water, donepezil hydrochloride, and whey protein hydrolysate (100 mg/kg), respectively. Model, WPH-L (low dose of whey protein hydrolysate), WPH-H (high dose of whey protein hydrolysate), and WP (whey protein) represent 20-month-old C57BL/6J mice treated with drinking water, the 10 mg/kg whey protein hydrolysate, 100 mg/kg whey protein hydrolysate, and 100 mg/kg whey protein, respectively, and 7-month-old C57BL/6J mice were given drinking water as the negative control group.

As illustrated in Figure 3A, the exploration time of the NOR test of ICR mice in the control group was less than that of mice treated with WPH (*p* = 0.015) and donepezil (*p* = 0.033), indicating that WPH consumption acts similarly to donepezil in enhancing aged mice’s capacity in exploration. Similarly, significant increases were observed for exploration time in aged 20-month-old C57BL/6J mice in the WPH-H (*p* = 0.001) and WP groups (*p* = 0.001), suggesting that WPH-H and WP improved memory as they spent more time at the new object (Figure 3B). During the shuttle test, the times that WPH-treated mice received electric shocks were reduced (*p* = 0.003) compared to the control group, showing a similar effect to donepezil (Figure 3C). Similarly, WPH and WP had a positive effect in alleviating memory impairment in aged C57BL/6J mice (Figure 3D). However, WPH-H possessed a more potent impairment-relieving capacity than WP (*p* < 0.001), which indicated that hydrolysis plays a vital role in either absorption, bioactivity or both in its memory enhancement property. Likewise, the step-down test on ICR and aged mice exhibited similar significant outcomes (Figure 3E,F).

Since the recognition index of the WPH-H and negative control group are similar, it might be concluded that the higher 100 mg/kg dose of WPH is necessary for a significant effect. Ni et al. [22] treated middle-aged mice with hydrolyzed chicken meat extract with significant improvements of NOR at 300 mg/kg. Yu [23] found that 1.5 g/kg whey protein peptides administered to aging C57BL/6N mice significantly increased NOR times. These comparisons might imply that the lower dose of WPH utilized in this study contains more bioactive elements that promote enhanced cognitive function. The 12-week human study of Kita et al. indicated that 1 g of whey peptides, including GTWY at 1.6 mg/d (about 0.23 mg/kg for 70 kg individual), could improve brain function [17]. In this 10 d study, the concentration of GTWY in WPH was 0.6% and the GTWY dose in a 100 mg/kg WPH-H dose would be 0.6 mg/kg. The equivalent human dose would be about 0.05 mg/kg according to Nair and Jacob [24]. Although our dose was lower and study time shorter, we speculated that GTWY and associated hydrolyzed whey protein peptides might play a crucial role in memory enhancement.

### 3.4. WPH Reduces the Oxidative Damage Stress in Mice with Memory Decline

**Figure 4:**
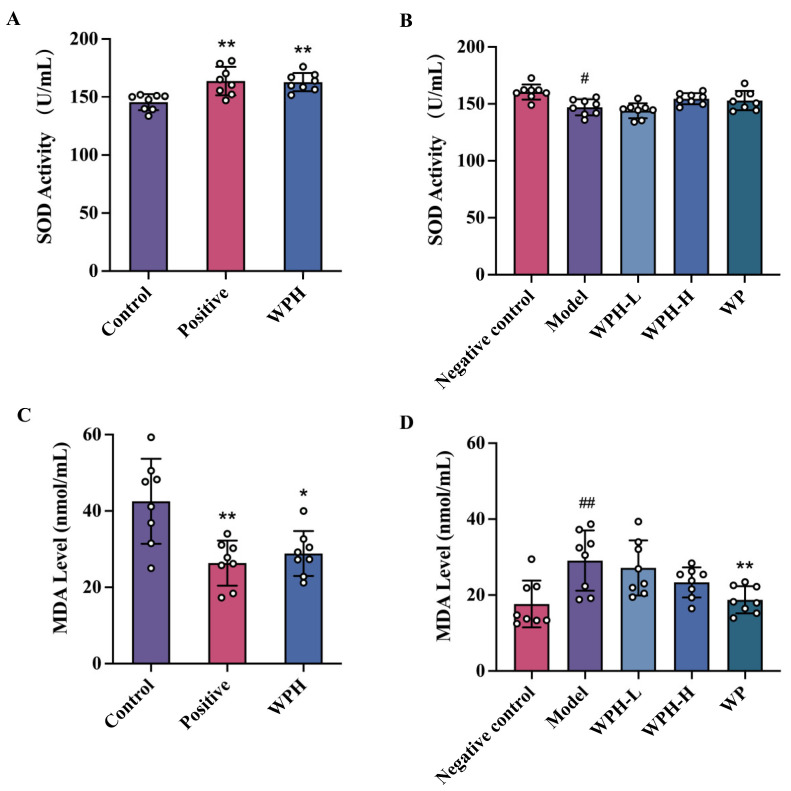
Effect of WPH and WP on oxidative stress of mice. SOD activity in serum of scopolamine-induced ICR mice (**A**) and old-aged C57BL/6J mice (**B**). MDA level in serum of scopolamine-induced ICR mice (**C**) and old-aged C57BL/6J mice (**D**). ^#^ means *p* < 0.05, and ^##^ means *p* < 0.01 for model group vs. control group; * and ** mean *p* < 0.05 and <0.01 for other groups compared to the model group in C57BL/6J mice and the control group in ICR mice. SOD, superoxide dismutase. MDA, malondialdehyde. Control, positive, and WPH represent ICR mice treated with distilled water, donepezil hydrochloride, and whey protein hydrolysate (100 mg/kg), respectively. Model, WPH-L (low dose of whey protein hydrolysate), WPH-H (high dose of whey protein hydrolysate), and WP (whey protein) represent 20-month-old C57BL/6J mice treated with drinking water, the 10 mg/kg whey protein hydrolysate, 100 mg/kg whey protein hydrolysate, and 100 mg/kg whey protein, respectively, and 7-month-old C57BL/6J mice were given drinking water as the negative control group.

To investigate the association between oxidative stress and cognitive decline, we measured the level of MDA and SOD in the serum (Figure 4A,B). The intervention of WPH significantly increased the serum SOD activity in scopolamine-induced mice (*p* = 0.0025), however, neither WPH nor WP had a substantial impact on aged C57BL/6J mice. As for MDA in the serum of scopolamine-induced mice, both donepezil and WPH interventions lowered their level by 37.13% (*p* = 0.003) and 35.71% (*p* = 0.008), respectively (Figure 4C). As shown in Figure 4D, MDA levels in the 20-month-old C57BL/6J mice (model group) were higher than those of the 7-month-old mice (negative control group). WP treatment caused an obvious decrease in 20-month-old mice (*p* = 0.005). Although there was a tendency for MDA levels in mice of the WPH-L and WPH-H group to decrease with concentration, the changes were not significant.

Although AD etiology and pathogenesis are not fully understood, increasing evidence from experimental and clinical data suggests that oxidative stress is a potential factor that should be considered in the progression of AD [25]. As signaling intermediates, reactive oxygen species (ROS) severely affect cell signaling. Their presence in brain tissue is associated with amyloid plaques or lipid peroxidation in the central nervous system. Many studies support the findings that oxidative stress detected in peripheral blood and plasma antioxidant levels are directly related to cognitive function [26]. In our study, WPH administration effectively decreased oxidative damage in mice with memory decline induced by scopolamine. The capacity of WPH to increase the activity of SOD and decrease ROS reactants such as MDA may be attributed to bioactive low molecular weight peptides in WPH, which would be consistent with previous studies [9,27].

### 3.5. Effect of WPH on AchE Level in Mice

**Figure 5:**
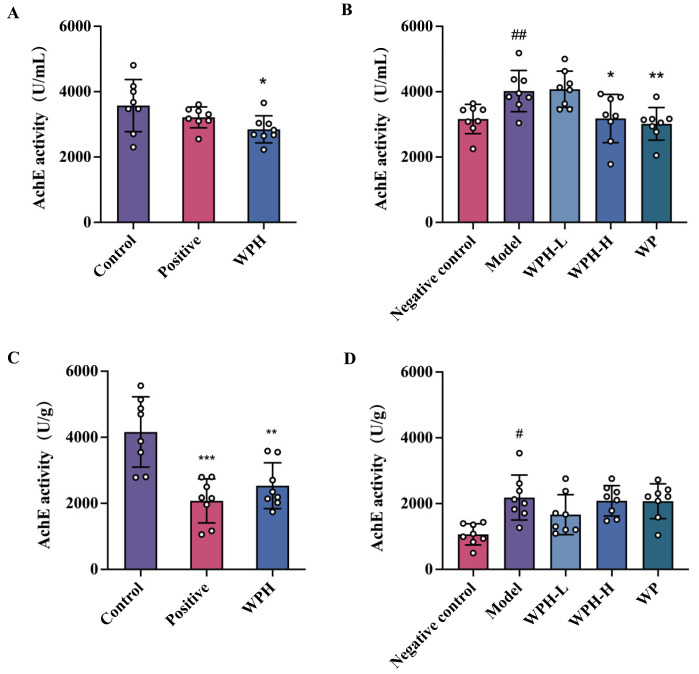
Effect of WPH and WP on AchE level in serum (**A**,**B**) and brain tissue (**C**,**D**); (**A**,**C**) scopolamine-induced ICR mice; (**B**,**D**) old-aged C57BL/6J mice. ^#^ means *p* < 0.05, and ^##^ means *p* < 0.01 for model group vs. control group; *, **, and *** mean *p* < 0.05, <0.01, and <0.001 for other groups compared to the model group in C57BL/6J mice and the control group in ICR mice. AchE, acetylcholinesterase. Control, positive, and WPH represent ICR mice treated with distilled water, donepezil hydrochloride, and whey protein hydrolysate (100 mg/kg), respectively. Model, WPH-L (low dose of whey protein hydrolysate), WPH-H (high dose of whey protein hydrolysate), and WP (whey protein) represent 20-month-old C57BL/6J mice treated with drinking water, 10 mg/kg whey protein hydrolysate, 100 mg/kg whey protein hydrolysate, and 100 mg/kg whey protein, respectively, and 7-month-old C57BL/6J mice were given drinking water as the negative control group.

The levels of AchE in the serum were demonstrated in Figure 5A. In ICR mice, WPH demonstrated better effects than donepezil in considerably lowering levels of AchE (*p* = 0.038). As for aged C57BL/6J mice, the levels of AchE in the model group were significantly higher than the young mice in the negative control group, while compared to the model group, the levels of AchE in the WPH-H and WP groups were significantly decreased to 20.87% (*p* = 0.028) and 24.95% (*p* = 0.003), respectively. As for AchE activity in brain tissue (Figure 5B), both donepezil and WPH treatments returned AchE to normal levels in scopolamine-induced mice. However, neither WPH nor WP significantly lowered AchE activity in the brain tissue of aged mice.

Acetylcholine is a crucial neurotransmitter in brain function; thus, its deficiency would lead to the dysfunction of nerve signal transmission. Based on the cholinergic theory, AChE level is highly related to memory impairment in AD. Additionally, AchE is considered to have a pro-aggregatory function to promote amyloid-*β* deposition [28]. In the present study, high AchE levels induced by scopolamine were lowered both in the brain and serum after WPH treatment and may attenuate related cholinergic system dysfunction. The effect of WPH and WP on the brain of aged mice was not apparent compared to that in serum, and whether this is related to age-related metabolic rate remains to be investigated.

### 3.6. Effect of WPH on Aβ_1-42_ Level in Mice

**Figure 6:**
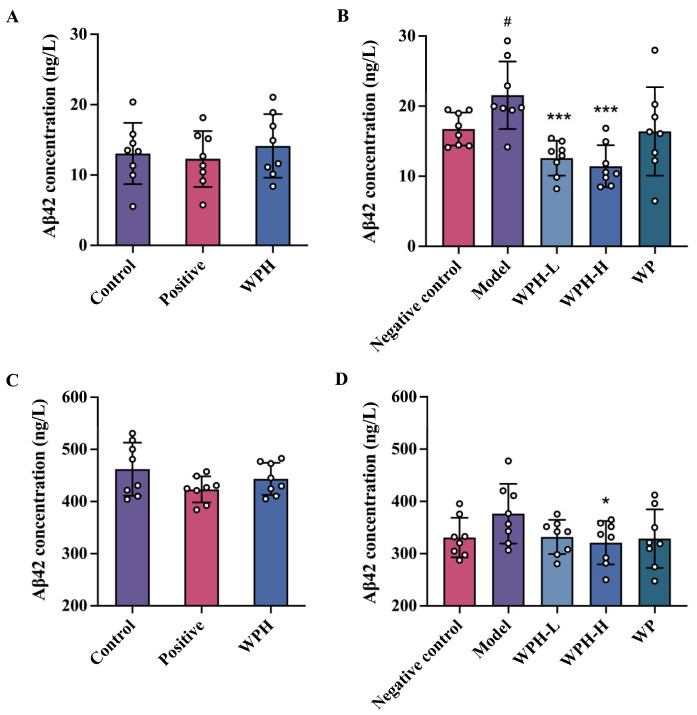
Effect of WPH on A*β*_1-42_ concentration in serum (**A**,**B**) and brain tissue (**C**,**D**); (**A**,**C**) scopolamine-induced ICR mice; (**B**,**D**) aged C57BL/6J mice. # means *p* < 0.05 for the model group vs. the negative control group. * and *** mean *p* < 0.05 and < 0.001 for other groups compared to the model group in C57BL/6J mice and the control group in ICR mice. A*β*_1-42_, amyloid beta protein 1-42. Control, positive, and WPH represent ICR mice treated with distilled water, donepezil hydrochloride, and whey protein hydrolysate (100 mg/kg), respectively. Model, WPH-L (low dose of whey protein hydrolysate), WPH-H (high dose of whey protein hydrolysate), and WP (whey protein) represent 20-month-old C57BL/6J mice treated with drinking water, 10 mg/kg whey protein hydrolysate, 100 mg/kg whey protein hydrolysate, and 100 mg/kg whey protein, respectively, and 7-month-old C57BL/6J mice were given drinking water as the negative control group.

Amyloid beta originates from the amyloid precursor protein in the brain. Research has shown that the level of Aβ_1-42_ in plasma could be a biomarker for mild cognitive impairment and correlates to the Aβ_1-42_ level in cerebrospinal fluid [29]. To investigate the impact of WPH and WP on Aβ_1-42_ in mice with memory deficit, we detected its concentration in serum and brain tissue, respectively. The results are shown in Figure 6. There was no significant difference in Aβ_1-42_ concentration in serum and brain after donepezil (*p* = 0.074) or WPH treatment (*p* = 0.393). No substantial change in Aβ_1-42_ concentration was observed in the brain between 7-month-old mice (negative control group) and 20-month-old mice (model group), but a high dose of WPH treatment significantly lowered Aβ_1-42_ level compared to the model group (*p* = 0.043) (Figure 6D). A noticeable reduction was also observed in serum Aβ_1-42_ level of aged mice in the WPH-L group (*p* < 0.001) and WPH-H group (*p* < 0.001) in Figure 6B. Kwon [30] also found a more evident reductive effect of Rhodiola sachalinensis extracts on serum Aβ_1-42_ levels than in the brain. A significant difference was shown at the dosage of 200 mg/kg. We suggest that longer intervention time and higher doses of WPH might be explored. Arendash [31] hypothesized that suppressing Aβ in the brain is associated with its transition between soluble and deposited forms. Then, the newly solubilized Aβ would be transported into the plasma. Now that we have observed the reduction of Aβ_1-42_ in the serum, it is plausible to presume that WPH treatment facilitates Aβ_1-42_ transport in brain tissue to improve cognition.

### 3.7. WPH Regulates the Gut Microbiome Relative to AD

**Figure 7:**
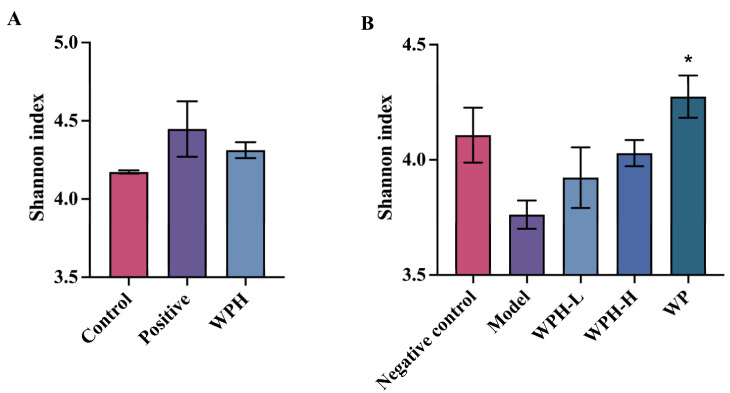

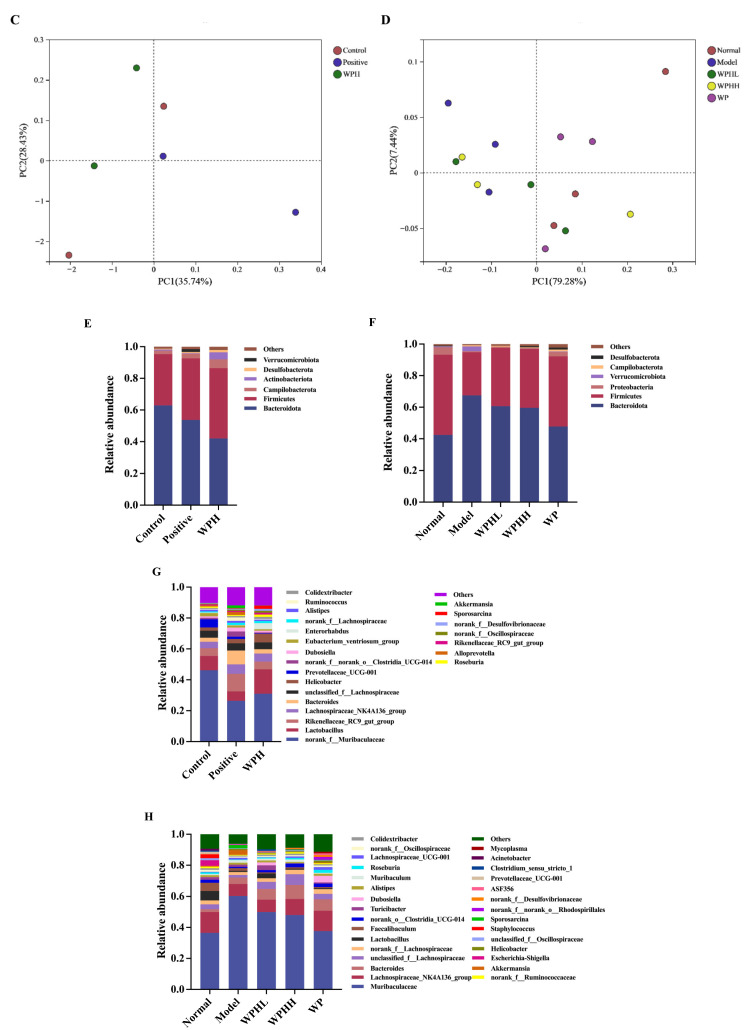
Effect of WPH and WP supplementation on gut microbiota structure in mice. Shannon index in α-diversity (**A**,**B**). Unweighted Unifrac distance-based principal coordinate analysis (PCoA) in β–diversity (**C**,**D**). Flora composition of each group at the phylum level (**E**,**F**). Flora composition of each group at the genus level (**G**,**H**). “Others” refers to genera with an average relative abundance of less than 1%. (**A**,**C**,**E**,**G**) Scopolamine-induced ICR mice; (**B**,**D**,**F**,**H**) aged C57BL/6J mice. * means *p* < 0.05 for other groups compared to the model group in C57BL/6J mice and the control group in ICR mice.

In recent studies, alterations in the gut microbiome with aging have been recognized for their contribution to the pathogenesis of AD due to the brain–gut axis [32,33]. Thus, bioactive substances might impact brain function by regulating the gut microbial community and diversity. As demonstrated in Figure 7A,B, the Shannon index of the alpha diversity in scopolamine-induced mice and normal 20-month-old mice (model group) is lower than that in other groups (*p* > 0.05), yet neither the pharmacological intervention nor the supplement of WPH resulted in significant improvements. Regarding beta diversity, the unweighted UniFrac distance of mice intervened by donepezil in the positive group differed from the control (Figure 7C,D). The community structure of aged mice was returned to a similar level as that in the 7-month-old mice (negative control group) after WP intervention.

As shown in Figure 7E, the proportion of Bacteroidota and Verrucomicrobiota was decreased, while Actinobacteriota was increased with WPH treatment. Verrucomicrobiota is considered a characteristic phylum of bacterial taxa between AD patients and healthy aged individuals according to Kaiyrlykyzy’s study [34], and Actinobacterota was reported to be related to the inhibition of AchE activity [35]. A similar composition was observed in aged mice (Figure 7G,H). In scopolamine-induced mice, the relative abundance of *Lactobacillus* significantly increased in the WPH group. Murray [36] reported that probiotics such as *Lactobacillus* had the effect of modulating memory dysfunction.

Subsequently, we further found that in ICR mice, WPH downregulated the abundance of Christensenellaceae at the family level and *ASF356* at the genus level (Figure S3). Barichela [37] found that increased Christensenellaceae were associated with cognitive impairment clinically, and it is reported that *ASF356* was elevated in mice with neurological damage [38]. Thus, these alterations brought by WPH may contribute to the promotion of mice’s cognitive ability.

### 3.8. Effect of WPH on the Hippocampus

**Figure 8:**
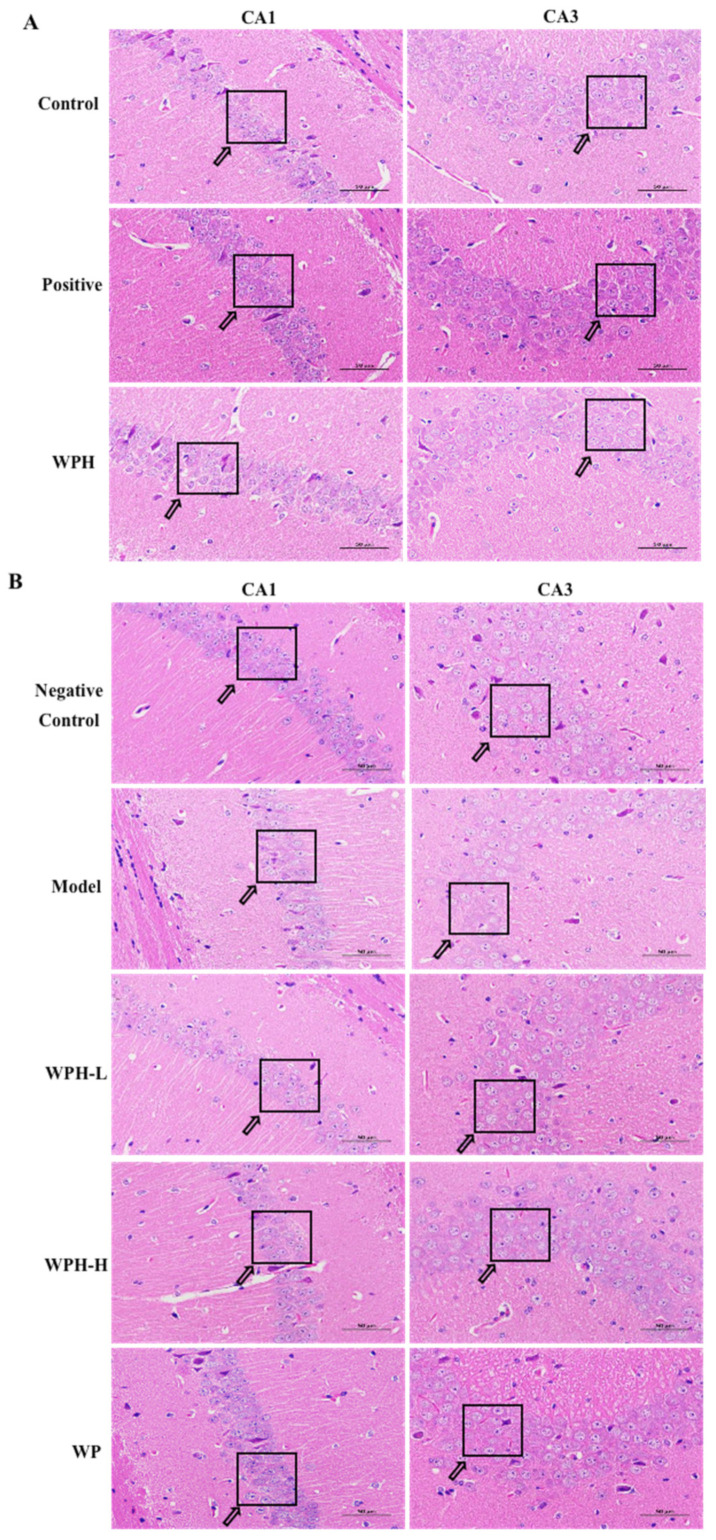
Pathological morphology of hippocampus in (**A**) scopolamine-induced ICR mice and (**B**) aged C57BL/6J mice. Scale bars = 50 μm. CA1 and CA3 indicate the corresponding region in the hippocampus. Control, positive, and WPH represent ICR mice treated with distilled water, donepezil hydrochloride, and whey protein hydrolysate (100 mg/kg), respectively. Model, WPH-L (low dose of whey protein hydrolysate), WPH-H (high dose of whey protein hydrolysate), and WP (whey protein) represent 20-month-old C57BL/6J mice treated with drinking water, 10 mg/kg whey protein hydrolysate, 100 mg/kg whey protein hydrolysate, and 100 mg/kg whey protein, respectively, and 7-month-old C57BL/6J mice were given drinking water as the negative control group. Arrows indicate the different states of cells.

HE staining of the hippocampus of ICR mice is shown in Figure 8A. Abnormally lesioned neurons occurred in the CA1 and CA3 regions after scopolamine injections (control), characterized by atrophy and disordered arrangement of nerve cells. In the positive group, the neurons with distinctive nucleolus morphology and clear nuclear membranes are neatly arranged compared with the control group. WPH treatment effectively alleviated scopolamine-induced neuronal damage in the hippocampus CA1 and CA3 regions, playing a neuroprotective role. Similar protective effects were noted in aged mice after WPH intervention (Figure 8B). The neural cells in the WPH-H group were closely aligned, and their pathological status was improved, indicating that the high dose of whey protein hydrolysates improved the hippocampal tissue status of old mice.

The hippocampus is critical to storing information and memory consolidation, depending on the functional and structural changes in specific fields [39]. Pyramidal neurons, also called place cells, are located in the CA1 and CA3 regions of the hippocampus, which plays a functional role in spatial navigation [40]. We found cellular damage in these two regions after scopolamine induction or when they entered old age. WPH reversed this damage by improving disordered arrangements and by reducing neuronal necrosis.

**Figure 9:**
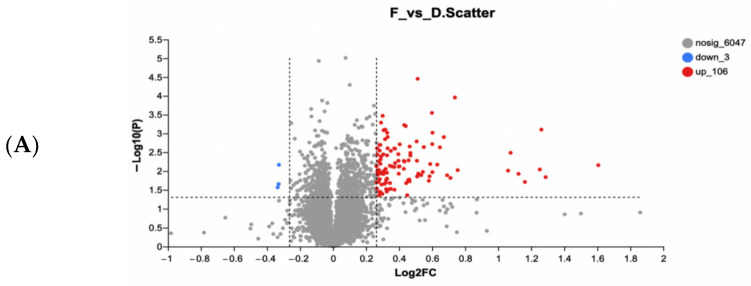

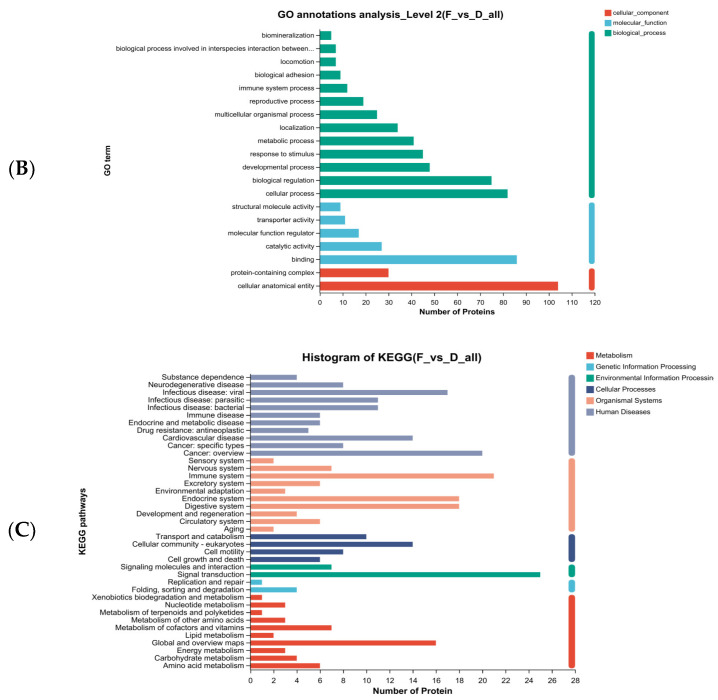
Volcano plot of DEPs from WPH-H group versus model group (**A**). The plot of differentially expressed proteins distribution in GO annotations analysis (**B**). KEGG pathway annotations analysis of differentially expressed proteins (**C**).

To further explore the mechanism of how the WPH improved memory, hippocampus proteomics was applied to identify the impact of WPH on protein expression in the hippocampus of aged mice. Figure 9A shows the distribution of all differentially expressed proteins (DEPs). To identify differentially expressed proteins (DEPs), fold change (>1.2 or 0.83) and *p* value (0.05) thresholds were utilized. Among 6156 proteins annotated, 106 proteins were upregulated, and 3 were downregulated after WPH treatment. Of the 106 upregulated proteins, four proteins, Ttr (FC = 2.44), Ppp1r1b (CF = 1.52), Rbp1 (FC = 1.50), and Car1 (FC = 1.35), were found that are cognition- and AD-related. The information about the molecular weight and peptide length of identified proteins was shown in Figure S4.

In serum and cerebrospinal fluid, transthyretin (TTR) can provide neuroprotection by binding Aβ to prevent aggregation [41]. Clinical research also shows that TTR concentration was lower in patients with AD [42]. Thus, highly expressed TTR would lead to lower Aβ levels in mice, which is remarkably consistent with our results in Section 3.6 (Figure 6). Ppp1r1b is a type of phosphoprotein that encodes DARPP-32 (dopamine and adenosine 3′5′-monophosphate-regulated phosphoprotein), which is involved in dopamine signaling and subsequently affects learning ability [43]. The upregulation of Rbp1 gene expression may contribute to memory enhancement by participating in retinoid metabolism. Retinoid X receptors (RXRs) play a vital role in nuclear receptor signaling. Rühl and colleagues [44] demonstrated that Rbp1 knockout mice display working memory deficits in the Y-maze test due to RXR-mediated signaling. Carbonic anhydrase 1 (Car1) is essential for signal processing and long-term synaptic transformation and could regulate GABAergic synaptic output [45]. Thus, we suspected that WPH improves the memory of aged mice through these proteomic changes (*p* < 0.05).

Functional annotation of DEPs using gene ontology (GO) analysis and cluster analysis is shown in Figure 9B. The GO enrichment analysis is shown in Figure S5A, and the detailed information is shown in Table S4. The results showed that DEPs were primarily located in the extracellular matrix and extracellular space. Evidence has accumulated that the hippocampus’s extracellular matrix (ECM) is related to neurological disorders such as age-dependent cognitive decline [46]. According to the KEGG pathway analysis result shown in Figure 9C, the pathways of signal transduction (25 proteins), endocrine system (18 proteins), and immune system (21 proteins) contained a high level of DEPs. The detailed description is shown in Table S5. The DEPs are primarily enriched in proteoglycans in cancer, focal adhesion, coagulation cascades, and ECM-receptor interaction (Figure S5B). Recent studies suggest that the focal adhesion pathway is one of the critical elements in AD pathogenesis involving β-amyloid peptide production and ECM communication [47]. Furthermore, Chen and Xia [48] found that complement and coagulation cascades have a link to Alzheimer’s disease. To conclude, we can speculate that WPH mainly participates in the ECM pathway, focal adhesion pathway, to perform a protective function on memory.

**Figure 10:**
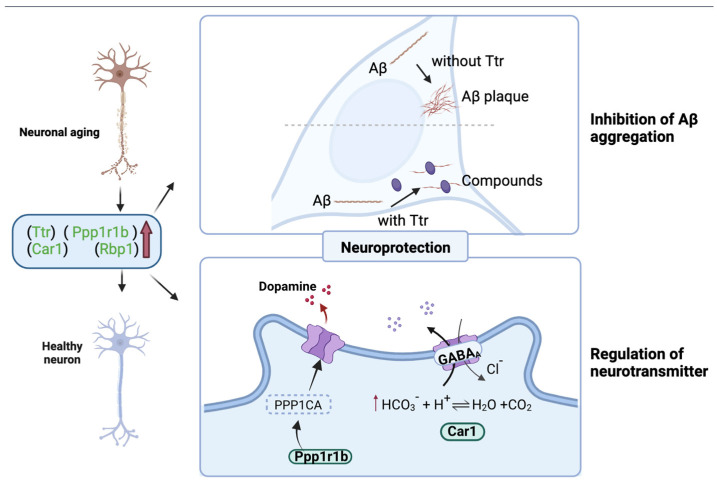
Schematic drawing of neuron protection and neurotransmission facilitation in cognitive and memory function enhancement mechanism.

The above analysis shows that WPH upregulates Aβ binding proteins and proteins associated with neurotransmitter signals, such as dopamine and GABA. The possible mechanism of WPH intervention is shown in Figure 10. On the one hand, it enhances memory by triggering the switch of synaptic state, and on the other hand, it protects neurons by reducing the aggregation of β-amyloid proteins. Therefore, WPH has neuroprotective properties and could promote cognition and memory.

## 4. Conclusions

This study demonstrated that short-time WPH and WP intake protected against memory deficit induced by scopolamine and aging. WPH intervention significantly improved the memory of aged and scopolamine-induced amnesia mice. The histopathological study of the hippocampus shows that WPH intervention alleviates neuronal damage. WPH enhances memory by triggering the switch of synaptic state, and it protects neurons by reducing the aggregation of β-amyloid proteins. Therefore, this study suggests the therapeutic benefits of WPH and provides a potential dietary management strategy for neurodegenerative diseases.

## Figures and Tables

**Table 1:** The effect of WPH on organic coefficients of mice in two models.

		Scopolamine-Induced ICR Mice			Aged C57BL/6J Mice				
		Control	Positive	WPH	Negative Control	Model	WPH-L	WPH-H	WP
Food intake (g/d)		5.50 ± 0.36	5.02 ± 0.24 *	5.25 ± 0.21	3.97 ± 0.53	4.56 ± 0.59	4.24 ± 0.43	3.95 ± 0.67	4.58 ± 0.25
Energy intake (kcal/d)		17.66 ± 1.14	16.11 ± 0.78	16.85 ± 0.66	12.74 ± 1.69	14.64 ± 1.90	13.61 ± 1.39	12.68 ± 2.15	14.70 ± 0.79
Organic coefficient (%)	Brain	1.25 ± 0.14	1.16 ± 0.11	1.17 ± 0.04	1.39 ± 0.18	1.09 ± 0.08 ^##^	1.13 ± 0.12	1.18 ± 0.14	1.18 ± 0.14
	Liver	4.42 ± 0.51	4.59 ± 0.10	4.50 ± 0.24	4.28 ± 0.29	4.52 ± 0.33	4.31 ± 0.33	4.71 ± 1.18	4.29 ± 0.63
	Heart	0.56 ± 0.09	0.48 ± 0.03	0.51 ± 0.05	0.61 ± 0.09	0.60 ± 0.09	0.54 ± 0.05	0.52 ± 0.04	0.61 ± 0.06
	Kidney	1.50 ± 0.19	1.47 ± 0.06	1.51 ± 0.11	1.37 ± 0.19	1.38 ± 0.21	1.33 ± 0.13	1.31 ± 0.15	1.27 ± 0.12
	Spleen	0.34 ± 0.04	0.35 ± 0.04	0.33 ± 0.04	0.29 ± 0.09	0.25 ± 0.05	0.22 ± 0.04	0.22 ± 0.11	0.27 ± 0.05

^##^ represents *p* < 0.01 for the model group vs. the negative control group; * represent *p* < 0.05 for the positive group compared to control group. The organic coefficient is calculated by organ weight (g)/body weight (g). Control, positive, and WPH represent ICR mice treated with distilled water, donepezil hydrochloride, and whey protein hydrolysate (100 mg/kg), respectively. Model, WPH-L (low dose of whey protein hydrolysate), WPH-H (high dose of whey protein hydrolysate), and WP (whey protein) represent 20-month-old C57BL/6J mice treated with drinking water, 10 mg/kg whey protein hydrolysate, 100 mg/kg whey protein hydrolysate, and 100 mg/kg whey protein, respectively, and 7-month-old C57BL/6J mice were given drinking water as the negative control group.

## Data Availability

The data that support the findings of this study are available from the corresponding author upon reasonable request.

## Supplementary Materials

The following supporting information can be downloaded at: https://www.mdpi.com/article/10.3390/nu15051228/s1, Figure S1: The scheme of animal experiment design; Figure S2. The proportion of parent protein for WPH and the peptide profile of β-Lactoglobulin in WPH; Figure S3. Relative abundances of genera that showed significant differences among groups at different taxonomic levels were obtained by one-way ANOVA. A represents scopolamine-induced ICR mice; B represents old-aged C57BL/6J mice; Figure S4. Protein molecular weight distribution (A) and peptide length distribution (B) in identified protein; Figure S5. Bubble chart of differentially expressed proteins in GO enrichment analysis (A) and KEGG enrichment analysis (B); Table S1: The mobile phase gradient of LC-MS/MS analysis; Table S2. The Gene Ontology (GO) enrichment information of WPH group and model group; Table S3. The KEGG pathway of differentially expressed proteins (DEPs).
