# Supplementary material for: Whey Protein Hydrolysate Renovates Age-Related and Scopolamine-Induced Cognitive Impairment
Source: Nutrients. 2023 Feb 28;15(5):1228. doi: 10.3390/nu15051228 (PMC10005054; doi:10.3390/nu15051228)
Supplement: Supplementary file 1 [file nutrients-15-01228-s001.zip › nutrients-2191651-supplementary.pdf]

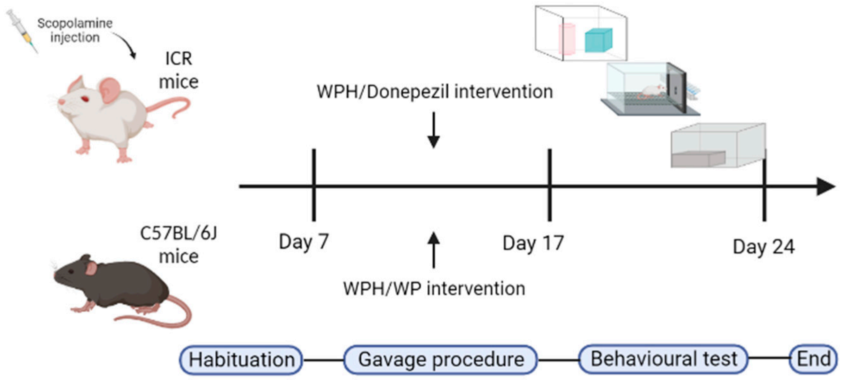

Figure S1. The scheme of animal experiment design.

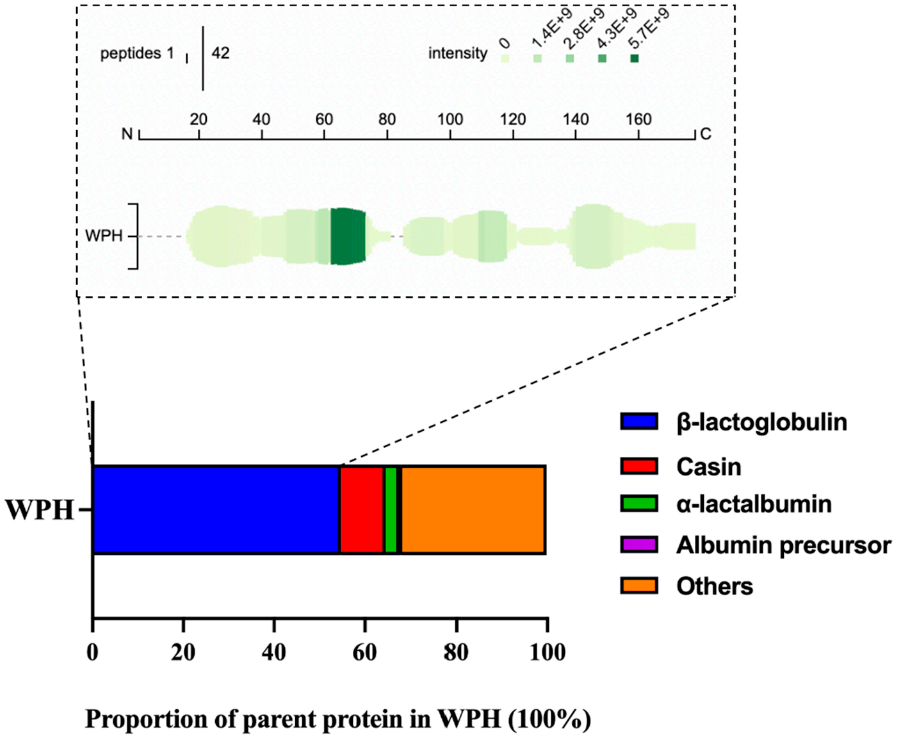

Figure S2. The proportion of parent protein for WPH and the peptide profile of β-Lactoglobulin in WPH.

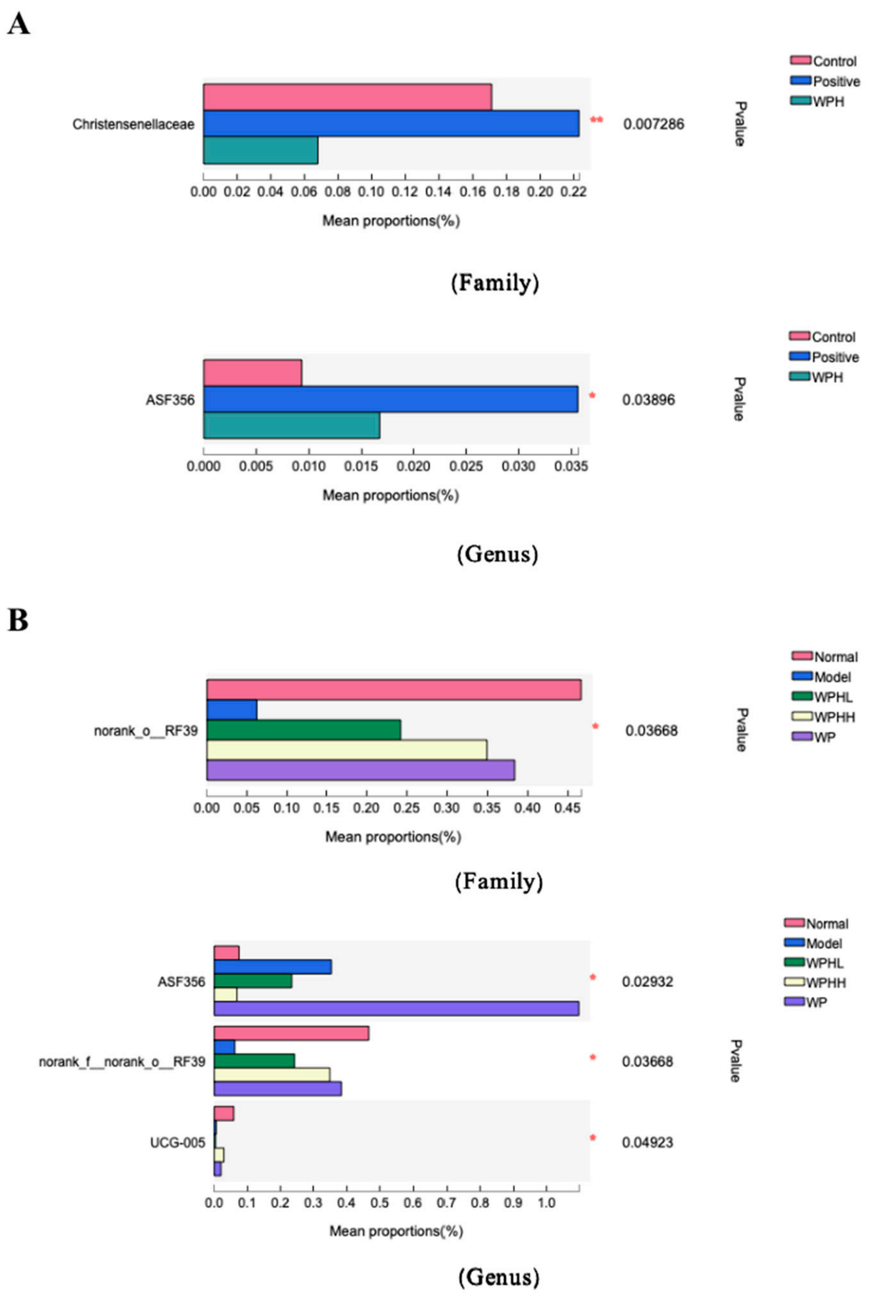

Figure S3. Relative abundances of genera that showed significant differences among groups at different taxonomic levels were obtained by one-way ANOVA. A represents scopolamine-induced ICR mice; B represents old-aged C57BL/6J mice. * and ** mean $p < 0.05$ and $p < 0.01$ compared to the model group.

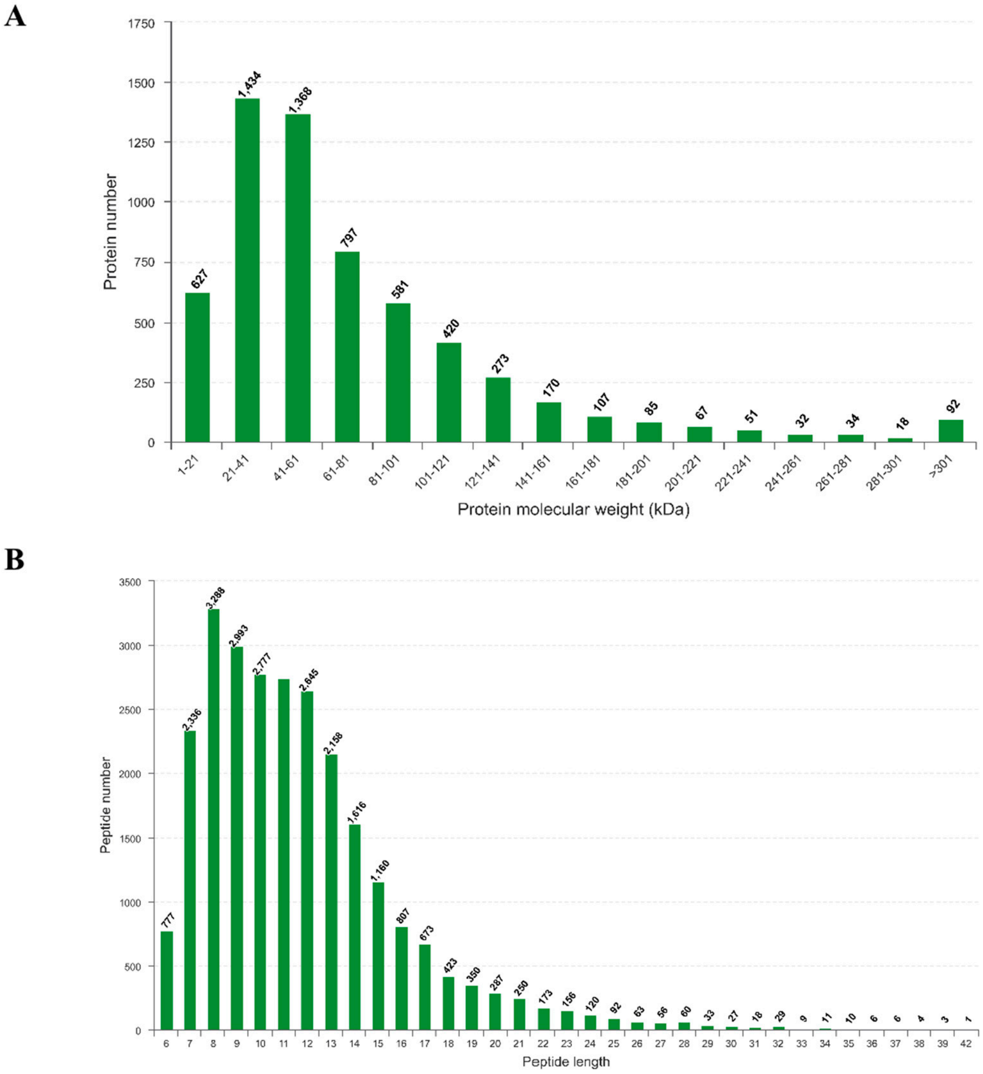

Figure S4. Protein molecular weight distribution (A) and peptide length distribution (B) in identified protein.

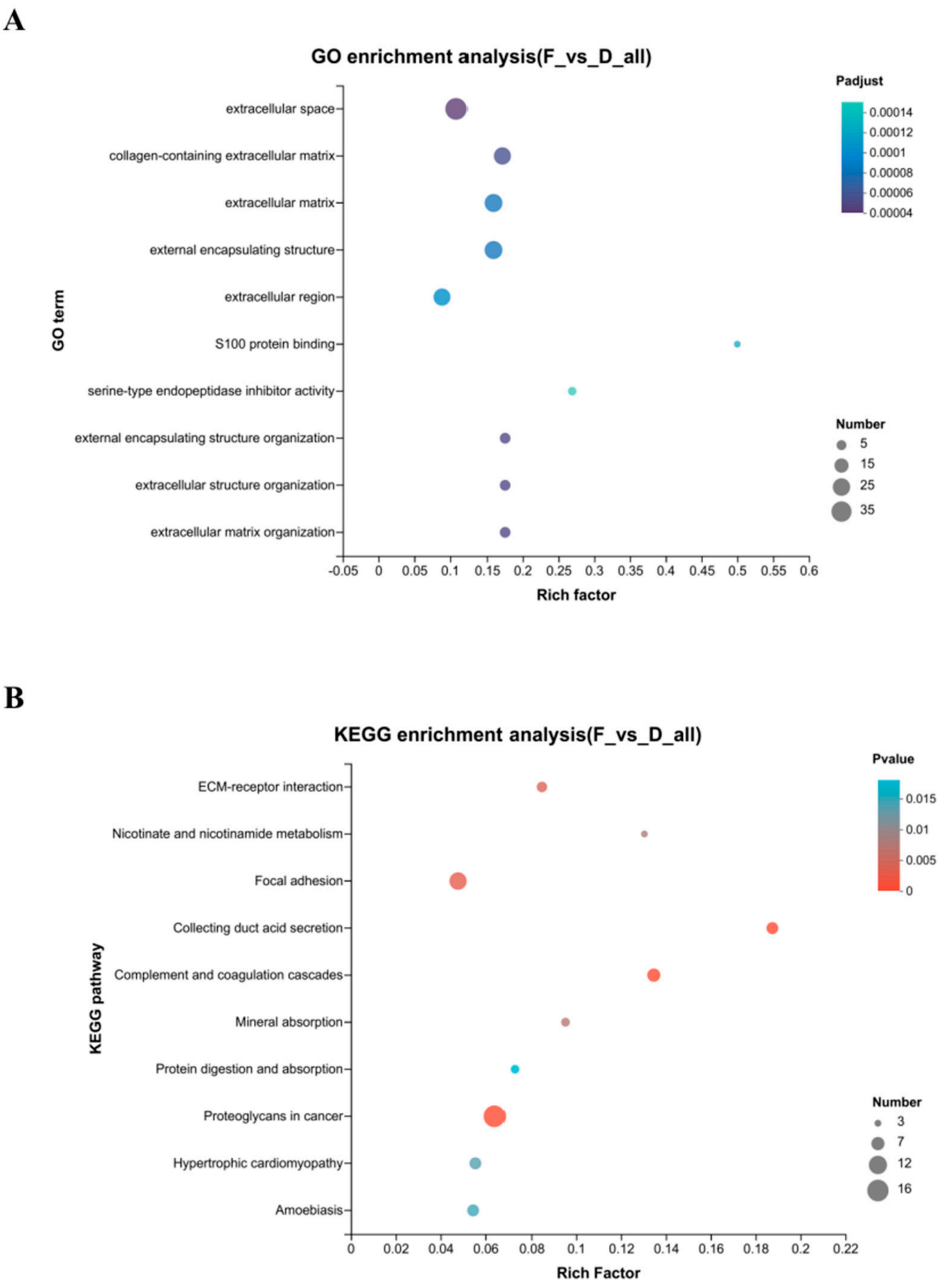

Figure S5. Bubble chart of differentially expressed proteins in GO enrichment analysis (A) and KEGG enrichment analysis (B).

Table S1. The mobile phase gradient of LC-MS/MS analysis

Time	Flow Rate (μL/min)	Solvent A (%)	Solvent B (%)
0	180	95	5
5	180	95	5
45	180	65	35
50	180	0	100
54	180	0	100
55	180	95	5
70	180	95	5

Table S2. The Gene Ontology (GO) enrichment information of WPH group and model group

**GO ID**	**GO Term**	**Category**	***p*-Value**	**Count**
GO:0005615	extracellular space	CC	0.000040535	35
GO:0045229	external encapsulating structure organization	BP	0.000049409	10
GO:0043062	extracellular structure organization	BP	0.000049409	10
GO:0030198	extracellular matrix organization	BP	0.000049409	10
GO:0062023	collagen-containing extracellular matrix	CC	0.000055591	23
GO:0031012	extracellular matrix	CC	0.000083263	25
GO:0030312	external encapsulating structure	CC	0.000083263	25
GO:0005576	extracellular region	CC	0.000099324	23
GO:0044548	S100 protein binding	MF	0.000122701	5
GO:0004867	serine-type endopeptidase inhibitor activity	MF	0.000145004	7
GO:0005201	extracellular matrix structural constituent	MF	0.000153598	5
GO:0050840	extracellular matrix binding	MF	0.000165506	6
GO:0004866	endopeptidase inhibitor activity	MF	0.000225268	8
GO:0030414	peptidase inhibitor activity	MF	0.000240522	8
GO:0061135	endopeptidase regulator activity	MF	0.00035769	8
GO:0002020	protease binding	MF	0.00035769	8
GO:0005604	basement membrane	CC	0.000370521	7
GO:0061134	peptidase regulator activity	MF	0.000817873	8
GO:0031214	biomineral tissue development	BP	0.000893933	5
GO:0110148	biomineralization	BP	0.000893933	5

Table S3. The KEGG pathway of differentially expressed proteins (DEPs)

KEGG Description	Pathway ID	DEPs Number	*p*-Value
Proteoglycans in cancer	mmu05205	16	0.000008589
Collecting duct acid secretion	mmu04966	6	0.000018824
Complement and coagulation cascades	mmu04610	7	0.000035606
Focal adhesion	mmu04510	11	0.00277315
ECM-receptor interaction	mmu04512	5	0.004074328
Mineral absorption	mmu04978	4	0.006696747
Nicotinate and nicotinamide metabolism	mmu00760	3	0.007851817
Hypertrophic cardiomyopathy	mmu05410	6	0.01315984
Amoebiasis	mmu05146	6	0.014311603
Protein digestion and absorption	mmu04974	4	0.017071603
